# Visual Interactions Conform to Pattern Decorrelation in Multiple Cortical Areas

**DOI:** 10.1371/journal.pone.0068046

**Published:** 2013-07-10

**Authors:** Fariba Sharifian, Lauri Nurminen, Simo Vanni

**Affiliations:** 1 Brain Research Unit, O.V. Lounasmaa Laboratory, School of Science, Aalto University, Espoo, Finland; 2 Advanced Magnetic Imaging Centre, Aalto Neuroimaging, O.V. Lounasmaa Laboratory, School of Science, Aalto University, Espoo, Finland; Centre national de la recherche scientifique, France

## Abstract

Neural responses to visual stimuli are strongest in the classical receptive field, but they are also modulated by stimuli in a much wider region. In the primary visual cortex, physiological data and models suggest that such contextual modulation is mediated by recurrent interactions between cortical areas. Outside the primary visual cortex, imaging data has shown qualitatively similar interactions. However, whether the mechanisms underlying these effects are similar in different areas has remained unclear. Here, we found that the blood oxygenation level dependent (BOLD) signal spreads over considerable cortical distances in the primary visual cortex, further than the classical receptive field. This indicates that the synaptic activity induced by a given stimulus occurs in a surprisingly extensive network. Correspondingly, we found suppressive and facilitative interactions far from the maximum retinotopic response. Next, we characterized the relationship between contextual modulation and correlation between two spatial activation patterns. Regardless of the functional area or retinotopic eccentricity, higher correlation between the center and surround response patterns was associated with stronger suppressive interaction. In individual voxels, suppressive interaction was predominant when the center and surround stimuli produced BOLD signals with the same sign. Facilitative interaction dominated in the voxels with opposite BOLD signal signs. Our data was in unison with recently published cortical decorrelation model, and was validated against alternative models, separately in different eccentricities and functional areas. Our study provides evidence that spatial interactions among neural populations involve decorrelation of macroscopic neural activation patterns, and suggests that the basic design of the cerebral cortex houses a robust decorrelation mechanism for afferent synaptic input.

## Introduction

Visual surroundings can change the neural response to a stimulus presented in the center of the receptive field. Such contextual modulation has been studied in cats [1]–[3] and monkeys [4]–[10]. In humans, contextual modulation has been studied with functional magnetic resonance imaging (fMRI), [11]–[13], and these results mirror the effects reported in psychophysics [14], [15]. In most cases context suppresses (i.e. decreases) the response, but facilitation have also been reported in behavioral [16]–[18], evoked potential [19], single cell [5], [10] and fMRI studies [11], [20], [21].

The network mechanisms underlying contextual modulation have been modeled with physiologically plausible models [22]–[24]. In these models, the feedforward-feedback loop integrates signals from spatially extensive areas of the visual field, thus providing the early visual areas access to information from large regions of the visual field. However, these models have not addressed the potential benefits of contextual modulation. Barlow [25], [26] and others [27]–[29] have suggested that reduction of redundancy of the output of a neural population, e.g. by removing correlations (decorrelation) between neural responses, is beneficial because it increases the efficiency of information transmission. Contextual modulation has been associated with efficient information transmission [30], [31] and indeed, context affects neuronal dependencies locally in the monkey primary visual cortex [32] and cat area 17 [1]. Vanni and Rosenström [21] studied contextual modulation and introduced a model in which contextual suppression and facilitation decorrelate not only local but also the macroscopic center and surround activation patterns in fMRI. Their cortical decorrelation (CD) model predicts that suppression and facilitation strength depends on signal correlation (overlap) between the center and surround fMRI activation patterns. However, it is unclear whether the same decorrelation process takes place in all areas of the visual cortex and beyond the subset of most active fMRI voxels. In addition, Vanni and Rosenström [21] did not validate their model against alternative and possibly better models.

The purpose of this study was to investigate how the correlation between spatial activation patterns induced by visual stimuli relates to suppressive (i.e. sublinear summation of center and surround response in case of combined center and surround stimulation) and facilitative (i.e. supralinear summation of center and surround response in case of combined center and surround stimulation) interaction strength. To address this issue, we studied how the BOLD signal spread and BOLD signal sign relate to contextual modulation in the visual cortex. Our results show that BOLD signals spread over large distances of visual cortex. In addition, our study generalizes the results of Vanni and Rosenström [21] and also validates such macroscopic pattern decorrelation model against linear and non-linear alternatives. Our findings are in line with the CD model and provide further evidence that pattern decorrelation is one of the computational roles of contextual modulation. The same match between the model and the data in different eccentricities and functional visual areas suggest that the spread of the BOLD signals reflect the synaptic input to postulated local mechanisms [33], [34], which modulate the neural responses and eventually reduce correlations between overlapping neural activation patterns.

## Methods

### Subjects

Fifteen subjects (age 20–44 years, 12 males) attended the experiment. All subjects had normal or corrected–to–normal vision, and gave their written informed consent before the measurements. The study was approved by the ethics committee of the Hospital District of Helsinki and Uusimaa.

### General Stimulus Design

Stimuli were produced with Matlab^TM^ (Mathworks, Natick, MA, USA) and Presentation^TM^ software (Neurobehavioral Systems, Albany, NY, USA) controlled the timing and positioning of the stimuli. The stimuli were projected with a 3–micromirror Christie C3™ data projector (Christie Digital System, Kitchener, Ontario, Canada) onto a back–projection screen in the magnet room. The subjects viewed the stimulus at 34–cm distance via a surface mirror in front of their eyes. Dim photopic background light was on during the experiments.

### Stimuli

**Figure 1:**
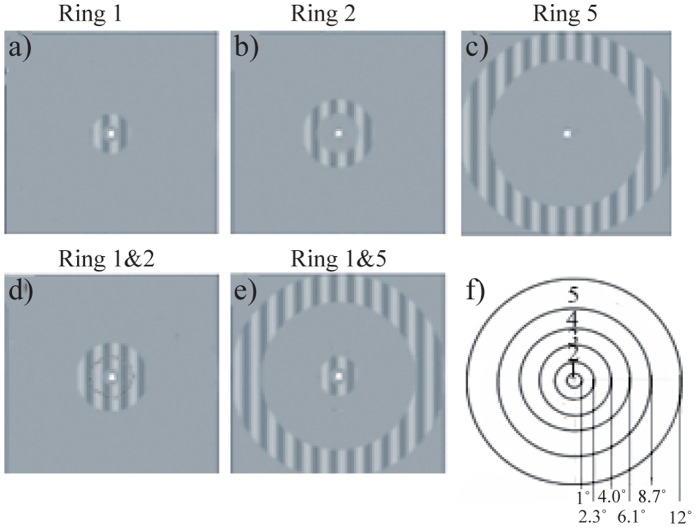
The main experiment comprised five different stimuli (a–e). a) Center (C, Ring1) alone, b) near surround (S_N_, Ring2) alone c) far surround (S_F_, Ring 5) alone d) center and near surround and e) center and far surround. The contrast of stimuli in this figure is higher than in the actual stimuli (15%). f) Eccentricity of the borders of the rings. The functional localizer for eccentricity representation comprised all five rings in a multifocal design.

Figure 1 a–e shows the 5 different stimuli where center, near and far surround were presented sequentially or simultaneously. The center and surrounds comprised a sinusoidal pattern at 0.5 c/deg spatial frequency. The contrast of the sinusoid was 15% and the stimuli were centered on the fixation. Display mean luminance was 40 cd/m^2^. The center stimulus was a 1.3 degrees wide ring extending from 1 to 2.3 degrees eccentricity (Ring1 in Figure 1).The near surround was 1.5 degrees wide and extended from 2.5 to 4.0 degrees eccentricity (Ring2). Thus a small gap always separated the center and the near surround stimuli. The far surround was 3.3 degrees wide extending from 8.7 to 12 degrees eccentricity (Ring5).

We functionally localized voxels of interest at different eccentricities in a separate run. The contrast of the checkerboard pattern reversed 8 times per second. The contrast of the pattern was 80%, and each ring included two rings of 24 identical radially expanding quadrilaterals. All the five rings (Fig. 1 f) were active in a multifocal design [35].

### Procedure

The order of the stimuli was pseudo–random and subjects were engaged in an attention control task during the stimulation. Four letters (Z, L, N and T) appeared at the fixation repeatedly for 150 ms each [36]. During the letter stream, 1–4 X-letters replaced some letters randomly and the subjects reported the number of X-letters in each block with a response pad during the rest period (only fixation). On average subjects identified correctly the number of X-letters in 73±12% blocks (no significant difference between stimulus and rest blocks, Friedman test, P = 0.43), which is clearly above the chance level (25%). The moderate performance suggests that the task was difficult enough to engage attention. Eye movements most probably do not constitute a major problem in our study, because the stimuli were symmetric around the fixation, the difficult task reduced the incentive for saccades, and our subjects were experienced. In general healthy subjects keep fixation with less than 10 arcmin accuracy [37].

The subjects participated in two separate sessions: functional visual area mapping experiment (described in more detail below) and the main experiment. The main experiment comprised five experimental runs and one functional localizer run. For each experimental run in the main experiment, 158 time points were acquired with 1.8 s repetition time, resulting in 4 minutes and 44 seconds duration. Each run comprised 30 blocks, and duration of one block was 9 seconds, including 6.75s stimulation (together with task at fixation, see above), and 2.25s rest periods. Separate task-only blocks with no stimuli enabled contrasting activation with a baseline condition where the task was comparable to active blocks. The functional localizer, acquired with the same volume as the main experiment, enabled individual definition of voxels of interest (VOI) separately for the five eccentricities. The functional localizer run had the same imaging parameters as the main experiment, but 164 time points, resulting in 4 minutes and 55 seconds duration. The functional localizer run comprised of 4 subsequences, each comprising 7 blocks of 9 sec duration. The temporal sequences of the five rings were mutually orthogonal resulting in linearly independent estimates of BOLD response from each ring (for details of the multifocal fMRI see [35]).

### FMRI Acquisition Parameters

FMRI data were acquired with General Electric Signa HDxt 3.0 T MRI (General Electric Medical System, Milwaukee, WI, USA) with an 8–channel phased array head coil. Five echo planar imaging runs were acquired for each subject. The acquisition matrix was 64*64, field of view 18 cm, resulting in 2.8 mm in–plane resolution, 29 slices, slice thickness 2.8 mm, 30 ms echo time and flip angle 60 degrees.

### Preprocessing

Data were analyzed with SPM8 (Wellcome Department of Imaging Neuroscience, London, UK) Matlab^TM^ toolbox. Standard preprocessing techniques, including image conversion to NIFTI format, slice time correction, and realignment with reslicing were applied before model estimation [38]. The first four images of each run were removed in order to keep only stable data. Freesurfer [39], [40] was used to visualize the signals on the inflated cortical surface. We followed the standard segmentation procedure, and an expert evaluated the quality of segmentation of each subject during and after the segmentation.

### Definition of Measured and Theoretical Modulation Coefficient (d)

We term the functional response in visual cortex to presenting the center stimulus alone as C, and surround alone as S (S_N_: Near surround and S_F_: Far surround). When the center and surround stimuli are presented simultaneously, the two patterns interact. In CD model we first measure the response pattern (voxel activation pattern) to center stimuli alone (C) and another response pattern to surround stimuli (S), both in the same set of voxels. It is not possible to measure the individual center and surround activity patterns when the center and surround are simultaneously presented. However, for modeling purpose we assume that the response to simultaneous presentation of center and surround is a sum of decorrelated center and surround activity patterns (Equation 1). The combined response is denoted by m(C,S) to emphasize that it is a measured (m) function of the original C and S activation patterns.
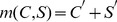
(1)


In equation 1 the C’ and S’ denote the decorrelated activity patterns.

Equation 2 defines *d* as a modulation index that indicates the quantity and sign (positive suppressive interaction, negative facilitative interaction) of contextual modulation. In our analysis we are dealing with two types of d values, *d_M_* which comes from the measurement (Equation 2) and *d_T_* which comes from the CD model, only based on C and S responses (Equations 3 and 4). The *d_M_* and *d_T_* are compared when evaluating the model. In practice, we first measure the C, S, and m(C,S) responses, and then calculate the *d_M_* coefficient for each voxel.
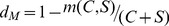
(2)


Thus, if there is no interaction between the component responses, *d_M_* equals zero. If response disappears totally (m(C,S) = 0), then *d_M_* is 1, indicating full suppressive interaction. If response comprise facilitative interaction so strongly that it is doubled (m(S,C) = 2 * (C+S)), then *d_M_* is −1. Although *d_M_* can take any real-value, it is likely that the most extreme values arise from noise; when the denominator C+S approaches zero, the result of Equation 2 approaches infinity. However we have not discarded the *d* values which are over 1 or below −1 in our results.

A single scalar theoretical *d*–coefficient (*d_T_*) (between [−1 1]) can fully remove correlation between any two vectors of voxel responses, C and S [21]:
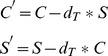
(3)


Equation 4 shows the formula for calculating the theoretical d in which var() is the variance function and cov() is the covariance function [21]. All correlation values are calculated with the Pearson’s formula (covariances divided by the product of standard deviations).
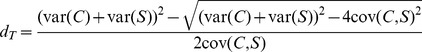
(4)


When studying the association of spatial correlation and neural interaction, we first selected a group of voxels (voxels of interest, VOI), whose BOLD signals for center and surround stimuli constituted the two response vectors. Next, the measured mean *d_M_* value for the VOI was compared to a theoretical *d_T_* value.

### Retinotopic Mapping, Calculation of Percent Signal Change and Selecting Voxels of Interest

Our stimuli in the main experiment activated V1, V2d, V2v and V3d in most subjects (average number of active voxels in each area was 270 in the right hemisphere). The borders of these areas were defined according to a 24–region multifocal localizer, adapted from the original 60–region stimulus [35]. Subjects attended a separate fMRI session for mapping of lower–order retinotopic areas (multifocal design of checkerboard contrast reversal stimuli) and ventral stream areas (block design with achromatic objects) in their right hemisphere [41].

The BOLD signal change was calculated as follows. The BOLD signal for the effect of interest was divided by the mean BOLD signal, separately for each run. Next we multiplied the quota with 100 to get the percent BOLD signal change. This analysis was repeated separately for each voxel, and then the values across the different voxels within a volume of interest were averaged.

In the main experiment we had five effects of interest: i) center, ii) near surround, iii) far surround, iv) center and near surround at the same time and v) center and far surround at the same time. These contrasts include implicit rest (not modeled with a separate regressor), i.e. each condition is contrasted to rest with one regressor.

All VOI selections are summarized in Table 1. We used three different ways to select the voxels of interest: 1) Voxels were selected based on active voxels for either the center, near surround, or far surround in the main experiment. This selection spans all visual functional areas in both hemispheres. The VOI_AVA_ was built from all five effects of interest together (Fig, 1 a-e). 2) Voxels were selected based on active voxels in multifocal functional localizer (P_FWE_ = 0.05, corresponding to T >4.7), providing representations for five different eccentricities (Fig. 1 f). 3) Voxels were selected from the main experiment stimuli, but assigned to distinct functional areas of the right hemisphere according to separate retinotopic mapping experiment. The active voxels were selected from contrast where all five regressors were on (see above). In practice, functional area labels on Freesurfer surface were intersected with the active voxels in the main experiment, which includes voxels active for the stimuli, but excludes voxels with just measurement noise.

**Table 1:** Description of voxels of interests.

VOI	Description
	**Voxels across all areas for the main stimuli**
VOI_C_	Center (Fig. 1 a)
VOI_SN_	Near Surround (Fig. 1b)
VOI_SF_	Far Surround (Fig. 1c)
VOI_AVA_	All visual areas
	**Voxels representing different eccentricities**
VOI_1°–2.3°_	Ring 1 of Figure 1e (1°–2.3° eccentricity)
VOI_2.3°–4.0°_	Ring 2 of Figure 1e (2.3°−4.0° eccentricity)
VOI_4.0°–6.1°_	Ring 3 of Figure 1e (4.0°−6.1° eccentricity)
VOI_6.1°–8.7°_	Ring 4 of Figure 1e (6.1°−8.7° eccentricity)
VOI_8.7°–12°_	Ring5 of Figure 1e (8.7°−12° eccentricity)
	**Voxels at different functional areas**
VOI_V1_	Area V1
VOI_V2d_	Area V2d
VOI_V2v_	Area V2v
VOI_V3d_	Area V3d
VOI_V3v_	Area V3v
VOIV_3AB_	Area V3AB
VOI_hV4_	Area hV4
VOI_VO_	Area VO
VOI_LO_	Area LO
VOI_V5_	AreaV5

Definition of all voxels-of-interest selections. See methods for details.

### Statistical Analysis

We used R–squared as a measure of goodness of fit between *d_M_* and the *d_T_*. As we had measurement errors in both correlation between center and surround responses and *d*–coefficient, we used orthogonal distances in calculation of the numerator in standard R–squared (Equation 5). The model error is the square of minimum distance from each data point to the model. The denominator of standard R–squared was calculated from the distance of each data point to the average of all data points.
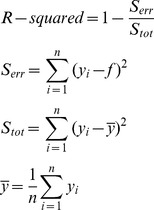
(5)


In Equation 5, n is the number of data points, y are the data points and f is the model.

The CD model can predict the modulation index (*d_M_*) by taking into account the pattern of activation for individual presentation of center and surround stimuli. However, we need to control weather any other model which relates correlation between center and surround responses to the modulation index can describe the data better than CD model. We defined four different models, which were compared with error measure. Our model had zero free parameters. The three other models were: i) a linear relation (with slope one and no offset from zero) between correlation between center and surround and *d_M_*, comprising zero free parameters, ii) a 3^rd^ order polynomial fit to each set of data point separately (Fig. S1) with four free parameters (a_1_–a_4_ in a 3^rd^ order polynomial function: Y = a_1_+a_2_X+a_3_X^2^+a_4_ X^3^) and iii) a linear fit to each set of data point separately with two free parameters (a and a_2_ in a linear function: Y = a_1_+a_2_X). For both polynomial fit and linear fit we used leave-one-out cross validation method to calculate the error. We took one data point out and made the fitting to all the remaining data points and measured the error from the fitted curve to the omitted point. Next we iterated the process across all the data points and then took the average of all these errors.

In addition to error measure we used Akaike information criterion (AIC) to compare relative goodness of fit of the mentioned models. The AIC takes the number of free parameters of each model into account as a punishment factor for the goodness of fit. In our analysis, AIC was calculated based on the least squared error of the data points [42] from the models.

Next, we compared the goodness of fit of the model for the local and global voxel selections. Because the total number of active voxels in each VOI affects the goodness of fit to the CD model, we resampled the data, in order to keep the number of tested voxels the same in different VOIs. Therefore we were able to compare fitting of decorrelation model (with no free parameters) in different functional visual areas and eccentricities. In resampling, 20 voxels were selected randomly from suprathreshold voxels within a VOI. Next, the difference between mean *d_M_* and *d_T_* was calculated to determine error between data and CD model. The procedure was repeated 100 times to determine the average error for both near and far surround within one VOI, and the whole procedure was repeated separately for each VOI.

In order to test if there is a significant suppressive interaction or facilitative interaction in group average data in different functional visual areas and in different eccentricities, we used sign–test with null hypothesis that data come from a continuous distribution with 0 median against the alternative that median is not 0.

We used the Mann–Whitney U test to analyze relationship between BOLD response sign and suppressive interaction and facilitative interaction. The null hypothesis of the test is that data in the two vectors (*d_M_* when C and S responses across voxels have the same signs and *d_M_* when C and S have different signs) are independent samples from identical continuous distributions with equal medians.

## Results

### Cortical Spread of BOLD Signal and Contextual Modulation

**Figure 2:**
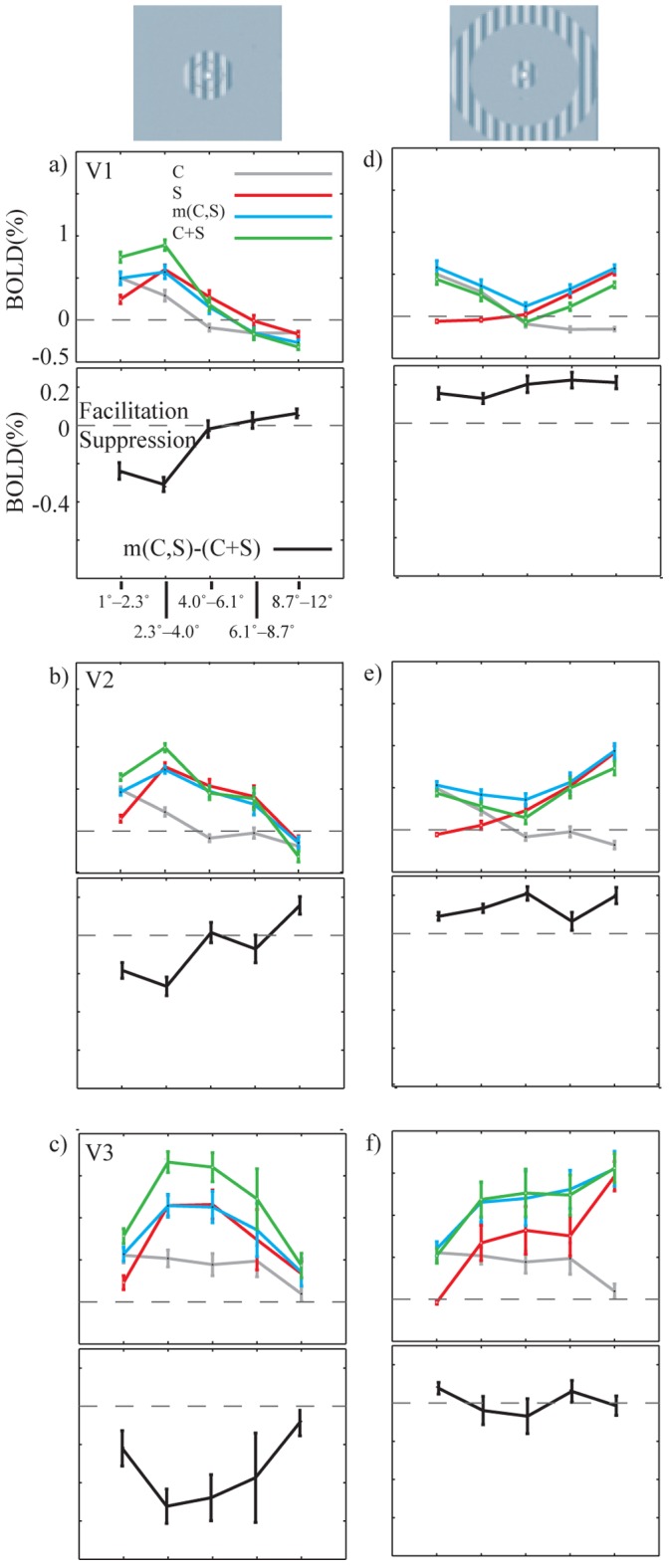
BOLD signal changes (%) for one subject (S3) as a function of eccentricity (VOI_1°_−_2.3°_–R_8.7°_−_12°_). The C, S, m(C,S) and C+S signals, for V1, V2 and V3 areas, are plotted separately for near surround (a–c) and far surround (d–f) conditions. The lower panel of each graph shows the subtraction m(C,S) – (C+S) revealing suppressive interaction or facilitative interaction amplitude. Voxel selection was thresholded at P_FWE_ = 0.05. Error bars display the standard errors of the means across active voxels. Note that the data for C is the same in the left and right columns.

We measured the BOLD signal at different eccentricities to analyze the BOLD signal spread along the cortex. Figure 2 shows the BOLD signal change (%) for near (Fig. 2 a–c) and far (Fig. 2 d–f) surround conditions for Subject 3. The positive BOLD signal spread significantly (Table S1) outside the above the threshold activation, i.e. the primary retinotopic representation, particularly in the V2 and V3. Significantly negative BOLD signals were also consistently found (Table S1) in voxels, which were far from the primary representation in the V1 and V2. This finding is consistent with earlier studies showing negative BOLD signals abutting the positive BOLD signals in cortical sensory areas, and corresponding reduction in neural activation when the BOLD signal was negative [43]–[45]. In line with previous single cell studies [10], the far surround produced less suppressive interaction than the near surround, or even facilitative interaction (+ in Table S1, in rows of m(C,S)-(C+S)), especially in the population of voxels where either the C or S or both responses were close to zero or negative (Fig. 2). In the example subject in figure 2 most such responses were found in V1 and V2.

**Figure 3:**
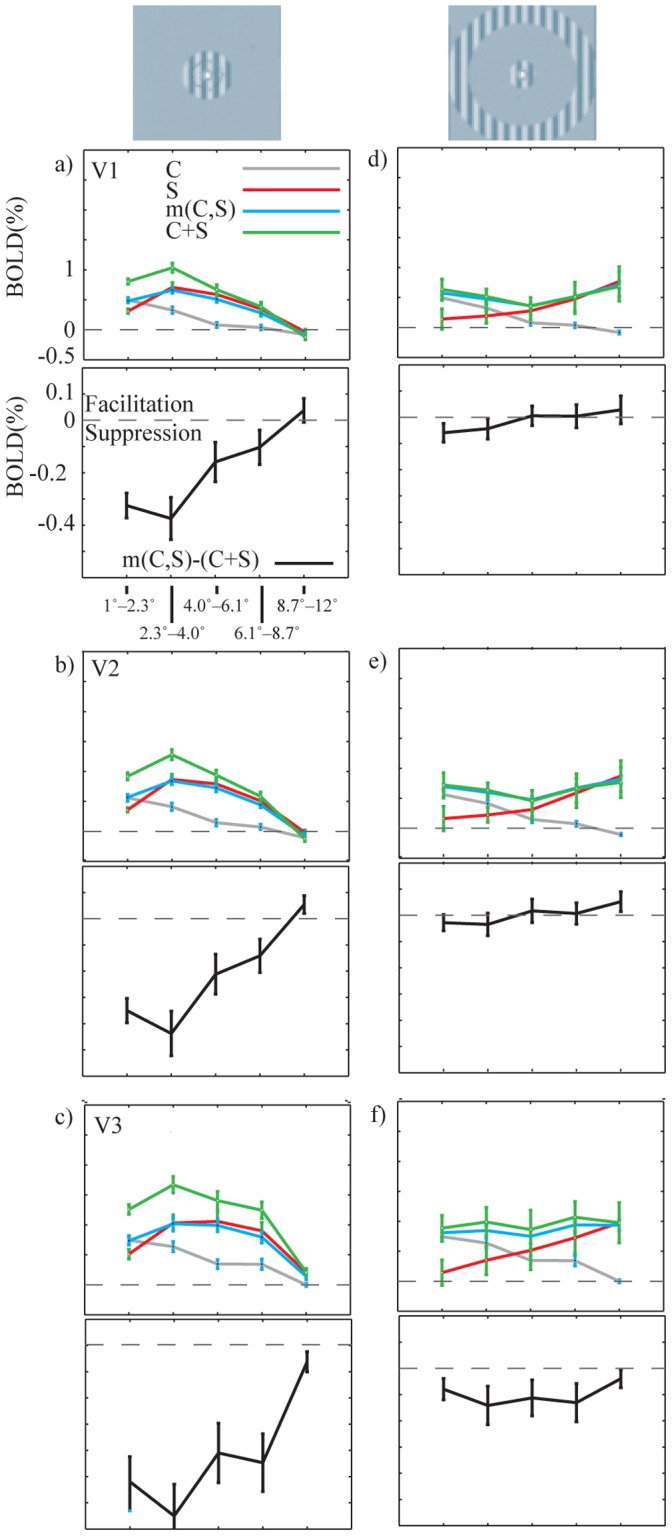
The group mean BOLD signal changes (%) as a function of eccentricity. See Fig. 2. for further explanation.

The group average followed the findings of Subject 3, and showed that the subthreshold BOLD signals spread significantly (Table S2) beyond the maximum retinotopic responses. Figure 3 shows the group average BOLD signal changes in cortical visual areas V1–V3, for representations of the five different eccentricities separately. From these data, we calculated at which eccentricity (in degrees) the BOLD signal crossed zero (Table 2, polynomial fit of 2^nd^ degree with 95% CI). In some cases no crossing took place in the sampled eccentricities, indicating positive signal spread to all sampled eccentricities. From the center to peripheral direction, the BOLD signal in V1 changed to negative value at cortical position which was representing 5.4° more peripheral part of the visual field than the outer edge of the center stimulus. The corresponding distances were 5.9° in the V2 and 7.8° in the V3. For near surround stimuli, in central direction, the BOLD was on average positive at the innermost ring in the V1, V2 and V3, corresponding to 1.3° more central representation than the inner edge of near surround stimuli; for peripheral direction from near surround, BOLD became negative on average at cortical position corresponding to 5.4° (V1), 5.7° (V2) and 6.4° (V3) more peripheral representation than the outer edge of the near surround. For far surround stimuli, in central direction, the BOLD remained positive over all sampled eccentricities in V1, V2 and V3, corresponding to 7.7° more central representation than the inner edge of far surround stimuli. Overall, these values are well in line with the mean BOLD signal spreads in V1, V2 and V3 in Zuiderbaan et al. [46]. We computed the cortical distances in V1 using the Schwartz formula [47]. The BOLD signals remained positive for distances that consistently exceeded 8 mm and in some cases (central direction, S_F_) even 28 mm (the red line in Fig. 3 d, upper panel). For the central stimulus, the BOLD signals consistently crossed to negative values in the most peripheral VOIs of V1 and V2 (Fig. 3 d and e, Table S2).

**Table 2:** At what eccentricity positive BOLD turns to negative BOLD.

			Center stimulus Peripheral direction,from 2.3°	Near Surround stimulus		Far Surround stimulusCentral direction,from 8.7°
				Central direction,from 2.3°	Peripheral direction,from 4.0°	
**V1**		**Average** **(95% CI)** **Degrees**	7.7°(3.8° – >12°)	<1°(2.3° – <1°)	9.5°(7.1° – 11.5°)	<1°(<1° – <1°)
		**Average** **spread** **(95% CI)** **Millimeters**	12.4 mm(2.3 mm –>19.2 mm)	>12.6 mm(5.6 mm –>12.6 mm)	8.5 mm(4.1 mm –11.4 mm)	>28.7 mm(>28.7 mm – >28.7 mm)
**V2**		**Average** **(95% CI)** **Degrees**	8.2°(4.6° – >12°)	<1°(3.2° – <1°)	9.7°(8.1° – 11.1°)	<1°(1.6° – <1°)
**V3**		**Average** **(95% CI)** **Degrees**	10.1°(4.7° – >12°)	<1°(<1° – <1°)	10.4°(9.5° – 11.2°)	<1°(<1° – <1°)

Spatial spread of positive BOLD signal for center, near surround, and far surround in V1, V2 and V3. The angles indicate the average (95% CI) eccentricity representation where the positive BOLD signal turns to negative BOLD signal. For central direction, the value at the title row indicates the inner edge eccentricity of the stimulus ring. For peripheral direction, the value indicates the outer edge, correspondingly.

Figure 3 suggests that when both the center and the surround stimuli evoked positive BOLD signal in the same voxels, suppressive interaction prevailed. Facilitative interaction was less frequent than suppressive interaction. In the group average fMRI, facilitative interaction was significant only in area V2 for VOI_8.7°–12°_ in S_F_ condition (p = 0.03, sign–test). Individual variation apparently reduced the significant facilitative interactions in group average data. At individual level, facilitative interaction was found in 9 subjects in 83 out of 450 various conditions (15 subjects* 5 rings * 3 functional visual areas* 2 near and far surround conditions = 450).

### Description of the Relationship between BOLD Signal Overlap and Decorrelation (*d*) Coefficient

**Figure 4:**
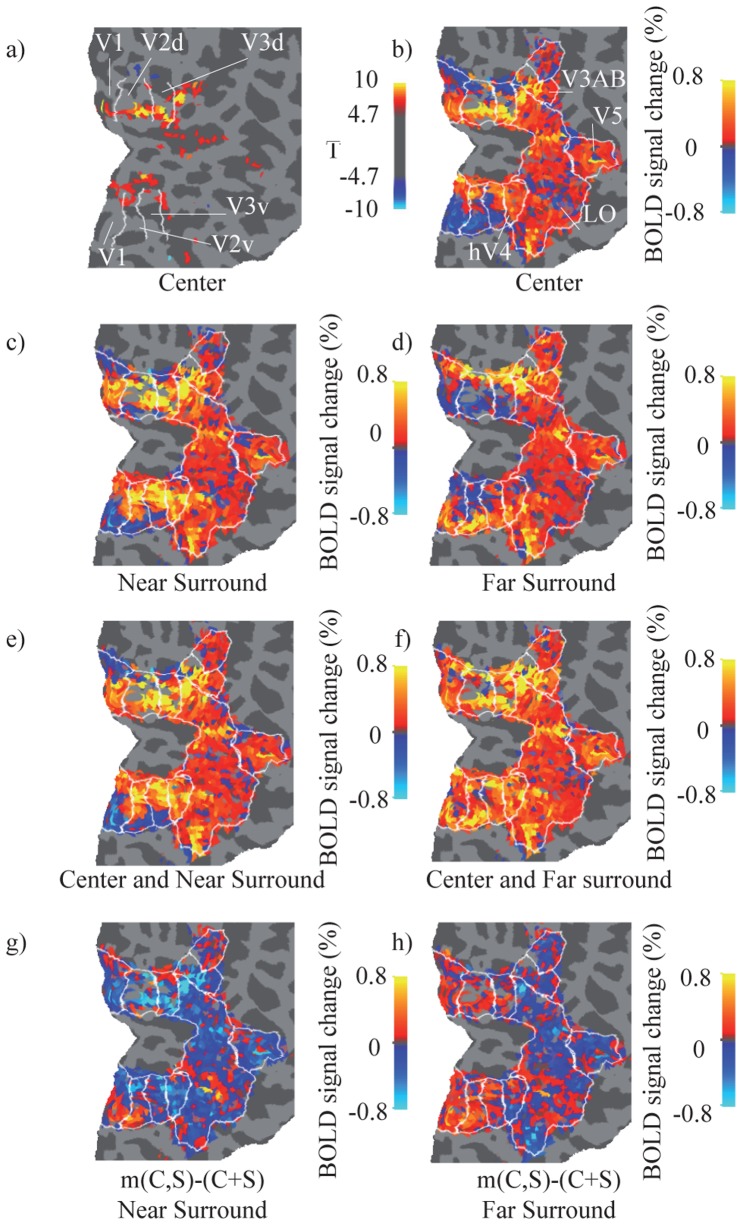
Relationship between BOLD signal overlap and interaction in Subject 3. a) SPM T–values (thresholded at P_FWE_ = 0.05, corresponding to T >4.7) for C contrast visualised on segmented and unfolded visual cortex. The white lines indicate the borders between visual areas. b) BOLD signal change (%) for C, limited to mapped functional visual areas. c) BOLD signal change (%) for S_N_. d) BOLD signal change (%) for S_F_. e) BOLD signal change (%) for C and S_N_ together (m(C,S_N_)). f) BOLD signal change (%) for C and S_F_ together (m(C,S_F_)). g) Difference of % signal change between m(C,S_N_) and sum of C and S_N_. A positive value (red-yellow) indicates facilitative interaction, and a negative value (blue) indicates suppressive interaction. h) Corresponding difference for m(C,S_F_) and C+S_F_.

**Figure 5:**
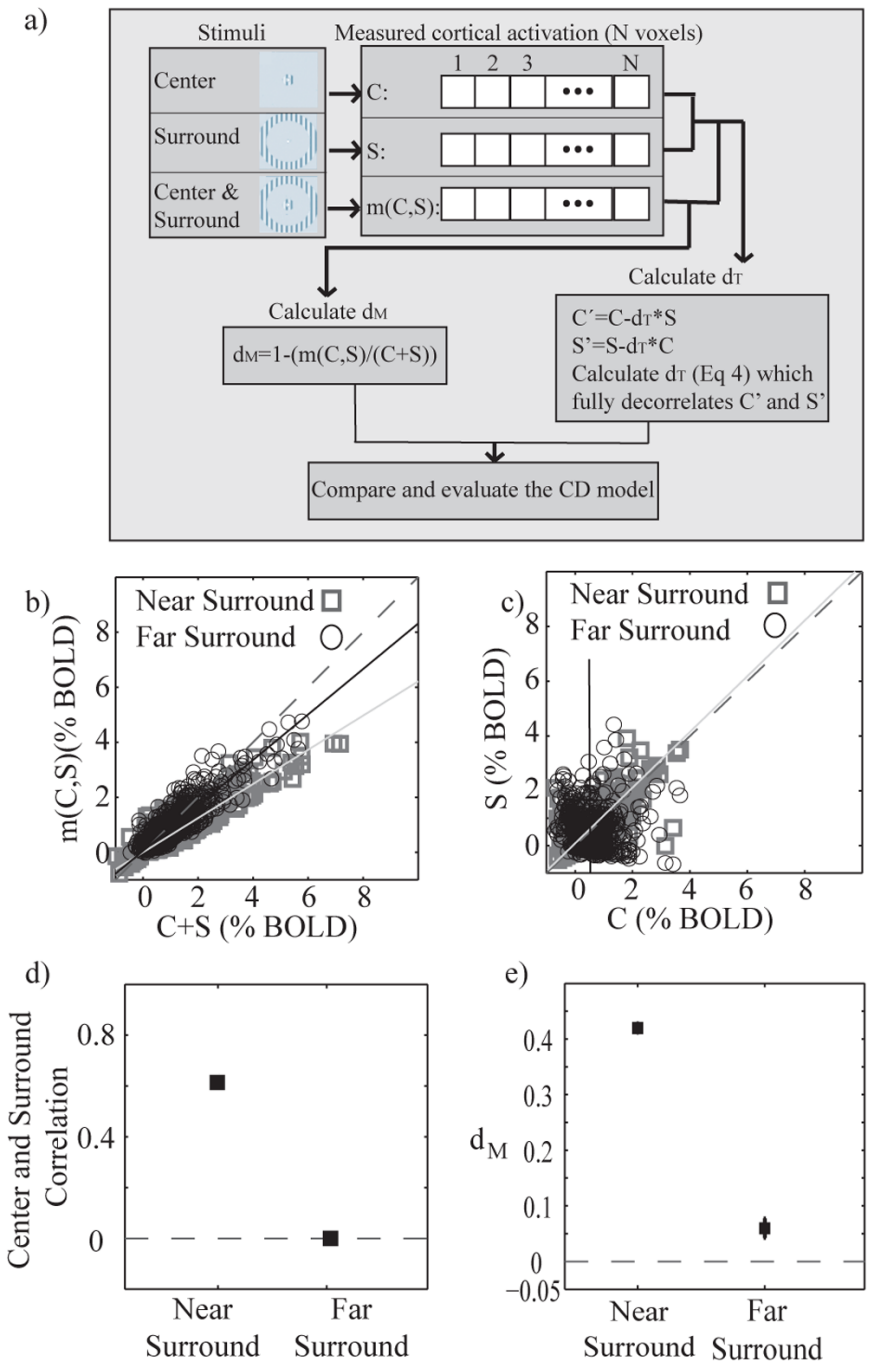
How to calculate theoretical and measured d and their relation with correlation of center and surround responses. a) Schematic view for calculating the theoretical and measured d (*d_T_* and *d_M_* respectively). b) m(C,S) signal change plotted as a function of C+S for the voxels of interests for the C stimulus (VOI_C_, P_FWE_ = 0.01). Black: C and S_F_. Gray: C and S_N_. The dashed line indicates the slope of one, and the solid lines linear regressors of the data. c) S signal change plotted as a function of C signal change for both near and far surround (VOI_C_, P_FWE_ = 0.01). The dashed line indicates the slope of one, and the solid lines linear regressors of the data. d) Correlations between C and S_N_ and C and S_F_ signals. e) Mean (SE) measured *d*–coefficient (*d_M_*) for both near and far surround condition (P_FWE_ = 0.01). BOLD % indicates BOLD signal change in %.

Figures 4 and 5 demonstrate how the patterns of activation for the C and S stimuli are correlated, and how this correlation is related to the decorrelation coefficient in the CD model. This part is intended to explain the nature of the decorrelation effect. The center stimulus evoked a clear activation in multiple functional areas (Fig. 4 a). As expected, the subthreshold positive BOLD response exceeded the area of thresholded activation, but the response turned consistently negative at sufficient distances from the thresholded activation in V1, V2 and V3 as described above (Fig. 4 b, Fig. 2). The data for near and far surround conditions were otherwise similar with the center only condition, but the activations were shifted according to the retinotopy (Fig. 4 c–d).

The BOLD signal change (%) for center and surround alone (Fig. 4 c–d), do not sum linearly when presented together (Fig. 4 e–f). Figure 4 g-h shows the difference between the simultaneous presentation and the linear sum, and reveals that the activation for center and surround presented simultaneously is not a simple sum of the components. Subtracting C+S from m(C,S) shows that the response to the combined stimuli may sum sublinearly (suppressive interaction; blue coloring in Fig. 4 g–h) or supralinearly (facilitative interaction; red–yellow coloring) relative to the unweighted sum of the components. The suppressive and facilitative interactions showed clear clustering in the cortex. Comparison of Figures 4 (b–h) shows that, especially in V1, V2 and V3, the facilitative interaction is found in most cases in areas of negative BOLD for either the center or surround stimuli, whereas suppressive interaction dominates where the response is predominantly positive. The BOLD activation patterns for the center, near surround, far surround (Figure 4 b–d) are significantly different with each other (Kolmogorov-Smirnov test, p<0.005). In addition, the interaction effect (the difference between m(C,S) compared to C+S) is significant (p<0.005) for both near and far surrounds (Figure 4 f–h).

Figure 5 a shows how *d_T_* and *d_M_* are calculated and Figure 5 b shows how m(C,S) BOLD signal deviates from the unweighted sum of C+S for all the voxels activated by the center stimulus (VOI_C,_ Table 1). The interaction effect (m(C,S)–(C+S)) was significantly different in near compared to far surround (Kolmogorov-Smirnov test, p<0.005). Clearly, there was more suppressive interaction when the surround was near the center (illustrated as larger distance from the slope of one), compared to when it was far from the center.

Figure 5 c and d shows that in this subject the spatial correlation (similarity of activation patterns) between C and S response vectors was positive when the surround was near the center (r = 0.62, p<0.01), and zero when the surround was far from the center (r = −0.02). The spatial correlation was defined as the Pearson’s correlation between the evoked cortical patterns for the center and the surround stimuli.

Figure 5 e shows the mean *d_M_*, which indicates the average measured modulation due to interactions. The near surround d is positive, indicating average suppression, and the far surround d is zero. In summary, when the correlation was high, the d was positive, and when the correlation was low, the d was close to zero, as suggested by the CD model [21].

### Group Analysis Suggests that Surround Modulation Decorrelates Overlapping Activation Patterns

**Figure 6:**
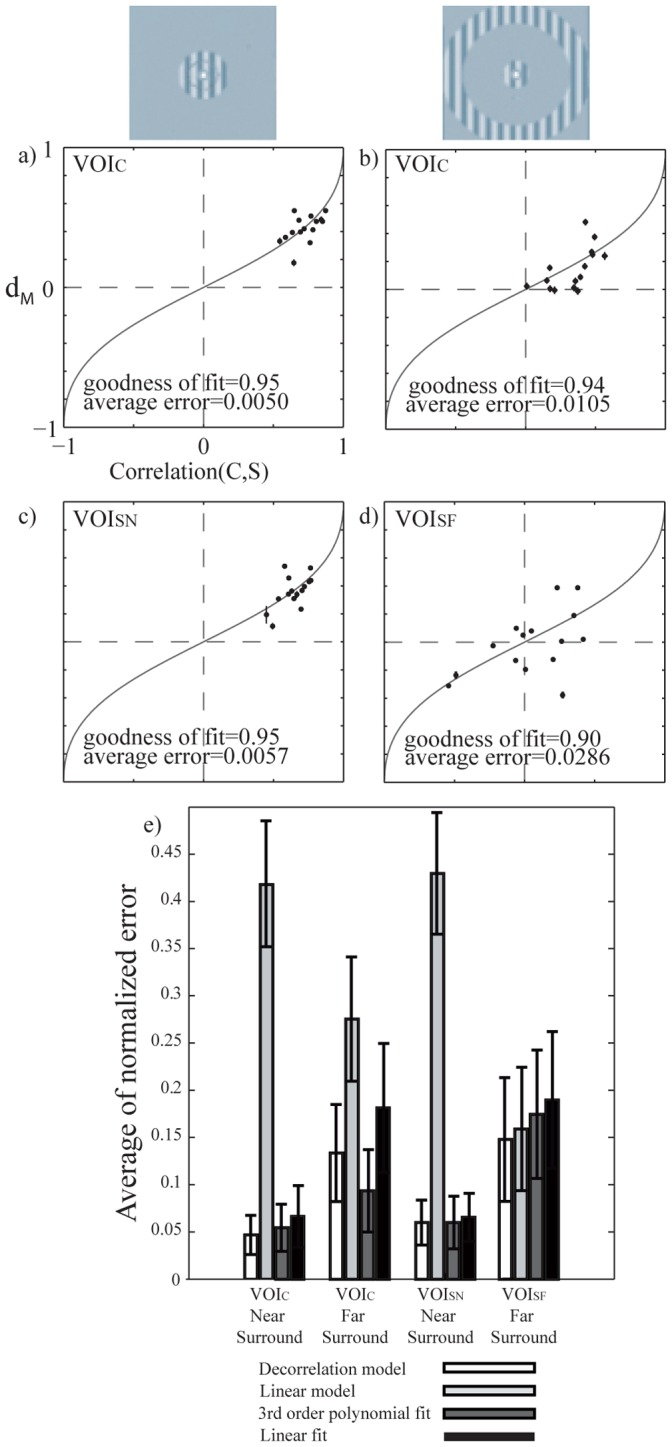
Correlation between C and S vectors versus average *d_M_*, for 15 subjects. The left and right columns show results for the near and far surround conditions, respectively. a)–b) VOI_C_ at P_FWE_ <0.01. c) VOI_SN_ and d) VOI_SF_. The error bars show standard error of means. The solid curves indicate the prediction (*d_T_*) from the CD model. The average error is the orthogonal distance from the model. e) Comparison of the four models, the CD model, linear model, 3^rd^ polynomial fit and linear fit. The average normalized error is calculated for four cases: in VOI_C_ for both near and far surround condition_,_ VOI_SN_ for near surround and in VOI_SF_ for far surround condition. In each VOI all the errors for individual data points are normalized to keep the maximum error in that VOI equal to 1. The error bars show standard error of mean for average error across all subjects.

We analyzed how correlation between center and surround mean response patterns relates to suppressive interaction and facilitative interaction in all subjects (N = 15). Figure 6 shows the individual mean *d_M_* values for near surround (left column) and far surround (right column) plotted as a function of correlation between center and surround (C,S) in the center VOI (Fig. 6 a–b), and in the near surround (Fig. 6 c) and far surround (Fig. 6 d) VOIs. The black curves in Fig. 6 a–d show the predicted *d_T_*. We have 15 (N subjects) * 4 (Fig. 6 a-d; VOI_C_ for near and far surrounds, VOI_SN_ for near and VOI_SF_ for far surround) = 60 correlation coefficients between the center and surround response patterns; in 56 data points out of 60 the correlation was significant (p<0.05). In VOI_C_ (average number of voxels 367) the data points cluster close to the model prediction (*d_T_*, solid curve) for both the near and far surround conditions. Although there are individual differences in distribution of *d_M_* for different subjects, the data follows reasonably well (goodness of fit ≥90%) the model prediction also for the near surround condition in the VOI_SN_, and for the far surround condition in the VOI_SF._ The far surround in VOI_SN_ and near surround in VOI_SF_ comprised no primary representation of either stimuli, which resulted in division with close to zero values in Eq. 2, and noisy d_M_ values. Therefore, the plots for the far surround in VOI_SN_ and near surround in VOI_SF_ are omitted for clarity.

In addition to our model which has no free parameters, we compared fitting of the data points to three other conceptually very simple models (see methods): linear relation between correlation of activation patterns and *d_M_* (linear model, no free parameters), 3^rd^ order polynomial fit (four free parameters) and linear fit (two free parameters). Figure 6 shows that CD model has significantly smaller average error compared to linear model (p<0.05, Friedman test) and linear fit (p<0.05). However no significant difference was found between the CD model and the 3^rd^ order polynomial fit (Fig. 6 e). In one case, the 3^rd^ order polynomial fit with four free parameters even finds the general form of the fixed-parameter decorrelation model when there are both positive and negative data points (Figure 6 d, Figure S1 d).

The four models are not directly comparable, because they have different numbers of free parameters. In a separate test, we compare AIC (see methods) of all the mentioned models, which takes into account the number of free parameters in a model. To be able do this, we calculated the least squared error for all the models in all the data points. Based on the AIC measure, the CD model has the highest probability to give the best fit among the existing models in VOI_C_ near surround (Fig. 6 a), VOI_SN_ near surround (Fig. 6 c) and VOI_SF_ far surround (Fig. 6 d). Our model was not the best model only in VOI_C_ far surround (Fig. 6 b) condition, where the 3rd order polynomial fit provided better fit to the data points. In summary, given the four different models and four conditions, our model was the best in 15/16 pairwise model comparisons. This indicates that our data is in unison with the decorrelation model.

### Relation between Measured and Theoretical Correlation Coefficient (d) at Different Eccentricities and at Different Functional Areas

To further analyze whether the CD model was more appropriate in one part of the cerebral cortex than another, we constructed two further voxel selections (Table 1), one along visual field eccentricity, and another for different functional areas. The null hypothesis was that there is no difference between voxel selections, which would mean that the decorrelation is similar everywhere.

**Figure 7:**
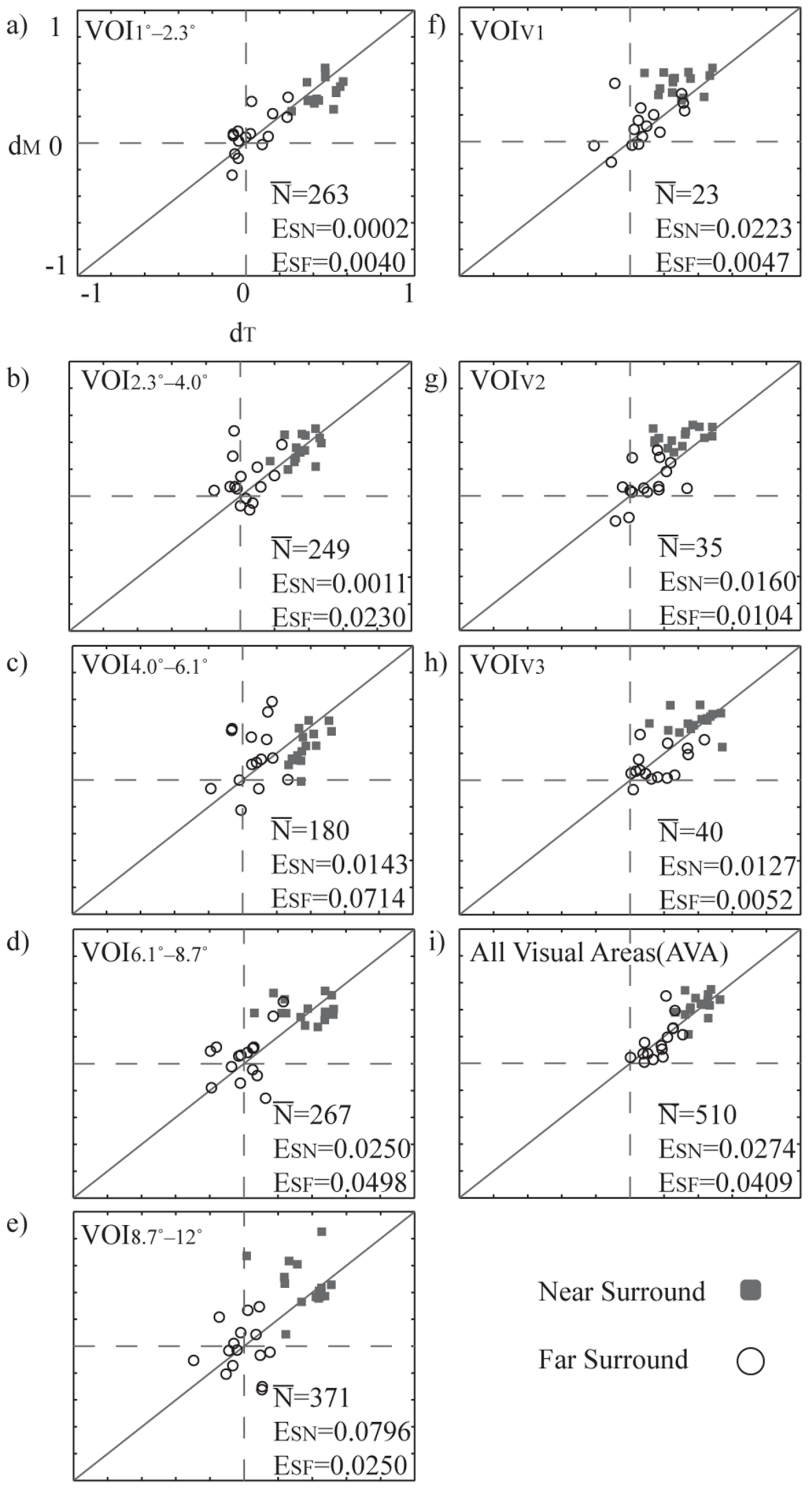
Optimally decorrelating *d_T_* values are plotted against median of measured *d_M_* values for all subjects, separately for near and far surround. a) – e) Eccentricities; VOI_1°_−_2.3°_–VOI_8.7°_−_12°_. f) – h) Functional areas; VOI_V1_, VOI_V2_, and VOI_V3_. i) All cortical visual areas (P_FWE_ = 0.01, VOI_C_). E_SN_ is the average squared error between *d_M_* and *d_T_* in near surround for the 100 sets of resampled (N = 20) voxels. A fixed selection size renders the errors independent from the number of voxels in one area. E_SF_ is the error for far surround. In each diagram, 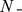 is the average number of supra–threshold voxels across subjects.

Figure 7 shows the relationship between *d_M_* and *d_T_* in different eccentricities (left column), and different functional areas (right column). All plots comprise data from both near (dark rectangles) and far surround (light circles) condition. In all areas we concatenated suprathreshold voxels from all visually responsive areas within one hemisphere. Figure 7 shows a particularly good match between *d_M_* and *d_T_* in VOI_1°–2.3°_, VOI2_2.3°–4.0°_ and VOI_AVA_ (a, b and i). However, because this match is confounded by the number of voxels in the selected areas, we conducted resampling with fixed number of voxels for quantification of the error in different VOIs. The average squared error (E, Fig. 7) after resampling is similar in all VOIs for both near (E_SN_) and far (E_SF_) surround representation.

We compared the error of the model in global (VOI_AVA_) selections of voxels to different local selections of voxels (Figures S2 and S3). We found no significant differences in errors between the local and global selection of voxels. The results were similar both for the different eccentricities (p = 0.1, separately for the near and far surround, Friedman’s test) and functional visual areas (S_N_, p = 0.6; S_F_, p = 0.5). For.

In summary, the strength of suppressive interaction and facilitative interaction follows the CD model [21] in all studied areas with similar accuracy.

### Response Signs Versus Modulation Sign

We found that the signs of signal changes (positive or negative BOLD) were linked to interaction sign (suppressive interaction or facilitative interaction). If both C and S activation signs were the same for a voxel, suppressive interaction was observed (Table 3). However, facilitative interaction was more common when the C and S were different in sign.

**Table 3:** Center and surround activation signs and contextual modulation.

	C and S withthe same activationsigns	C and S withdifferent activationsigns
N_Suppression_d_M_ >0	862	109
N_Facilitation_d_M_ <0	188	219

Average number of voxels (VOI_AVA_) across all subjects showing facilitative interaction (N_Facilitation_) and suppressive interaction (N_Suppression_ ), when center (C) and surround (S) had the same and when they had different sign of BOLD response.

Mann–Whitney U test confirmed this qualitative finding. Most (87%) of the subjects rejected null hypothesis (that data in the two distributions are independent samples from identical continuous distributions with equal medians, see method) for the near surround (at p<0.001) and all the subjects rejected null hypothesis for the far surround (at p<0.001) interactions. This indicates that suppressive interaction and facilitative interaction are associated with relative signs of center and surround responses.

## Discussion

We studied contextual modulation with fMRI and our results can be summarized as follows: i) In lower–order retinotopic areas, where we expect local activation for local stimuli, BOLD signal change as well as contextually induced suppressive and facilitative interactions emerged far from the maximum retinotopic response. ii) The CD model [21] was consistently better than the other models in predicting the relation between correlation and interaction strength. iii) The CD model performance was similar in all studied visual areas. This suggests that similar mechanisms underlie these effects in all visual cortical areas, and that the same model can be generalized outside the most active voxel population. iv) Suppressive interaction was associated with different signs of center and surround mean BOLD responses; in contrast, facilitative interaction was associated with center and surround BOLD responses of the same sign.

### Cortical Spread of BOLD Signal and Contextual Modulation

Our results showed contextual effects over very long distances in the visual field. This is in line with a recent report by Haak et al. [48] who showed that population receptive fields are significantly displaced if the stimulation is limited to one part of the visual field. These contextual effects follow the long-distance spread of BOLD signal increments and decrements around the maximum retinotopic response (Table 2). The positive BOLD signal spread exceeds 8 mm in V1 and in some cases more than 28 mm, which is obviously more than the radius of horizontal connections (∼ 3 mm) in monkey V1 [8]. These values are consistent with distant (7.15+/−3.14 mm away from the nearest positively responding area in V1) negative BOLD signals that have been found in monkeys [43], as well as with earlier reports of the surround sizes of fMRI population receptive fields [46].

The wide–spread BOLD signals are in line with earlier findings of the very long–range spatial interactions in single V1 neurons [10] and in human contrast perception [18], as well as with earlier imaging data of distant surround modulation in human visual cortex [49], [50]. These widespread signals may reflect the anatomical substrate which connects local neural populations to functional networks. In recent network models [23], [24] and in an earlier *first–pass* model (for a review, see [51]), such long–range interactions arise from the very rapidly conducting feedforward–feedback loops between the primary visual cortex and extrastriate areas. In the *first-pass* model, neurons in extrastriate areas integrate signals from large areas of the visual field, and then feed back their output to primary visual cortex. This results in modulation of neuronal responses in the primary visual cortex, which is caused by stimuli far away from the classical receptive field. After receiving these signals, local mechanisms may be enough to carry out the computations which result in the modulation of single neuron responses [33], [34].

### Comparison to Earlier Models and Neural Data of Contextual Modulation

We observed the strongest suppressive interaction when the correlation between center and surround activation patterns was the highest. Moreover, the theoretically computed fully decorrelating modulation index *d_T_* was in good harmony with the measured values in nearly all studied eccentricities and visual areas. These findings indicate that the same decorrelation principle sets the strength of both facilitative and suppressive interaction across visual areas. The same decorrelation principle probably holds for other types of stimuli as well, as it has been tested earlier with faces and objects [21]. Similar effects have been observed electrophysiological studies, with contextual modulation decreasing redundancy of neural responses in the primary visual cortex of monkeys [31] and the area 17 of cats [1]. It is interesting that also in the olfactory system of zebrafish, decorrelation of activation patterns emerges when stimuli are presented simultaneously [52], [53]. Together with previous evidence [1], [32], [52], [53], our study suggests that contextual modulation affects not only the manner in which single neurons encode sensory stimuli, but also the encoding by a population of neurons. This, again, is reflected in the macroscopic pattern of activation, which can be measured with fMRI. It is not surprising that we found decorrelation in the macroscopic activation patterns, given that decorrelation has been found in single neurons in the primary visual cortex [30], and that macroscopic activation patterns comprise significant information about cortical representations (reviewed in [54], [55]).

Standard single cell [9], [56] and psychophysical [17], [57] models of contextual modulation incorporate some type of divisive gain control mechanism. These models relate closely to models of divisive normalization [58], [59], which describes the effect of context on the contrast response of single cells. Although our assumption of subtractive interaction is at odds with the models assuming divisive interaction, a close inspection of the single cell data by Cavanaugh et al. [9] reveals that the interaction is actually subtractive in a large proportion of V1 cells. Moreover, while the gain control models describe the effects of context on the response of a single neuron or on a psychophysical mechanism, the CD model describes contextual modulation at the neural population level, with no parameters derived from the stimulus.

In addition to divisive normalization, Max model [60] has been used to explain neural interactions in particular cortical neurons, and Average model [61] has reliably predicted the neural interaction in inferotemporal cortex. Max model estimates the response to a complex stimulus as the maximum response of the components, whereas the Average model estimates the response as the average of the components’ responses. These two models can be quantitatively compared to the CD model by calculating their prediction error. The CD model is in correspondence with our BOLD responses to the combination of center and surround stimuli (m(C,S)) better than the AVERAGE model (t-test, p = 0.004, comparison of the norm of the prediction error for center and near surround and center and far surround interactions, for all the 15 subjects, voxel selections match the four panels in Fig. 6). In contrast, the CD model did not significantly differ from the MAX model (t-test, p = 0.4); thus a more thorough comparison between these two models is required in future studies, for example by varying the response strengths of the component patterns systematically.

Finally, it is worth noting that fMRI is most sensitive to synaptic activity [62]; in combination with the nonlinear mapping of membrane voltage change to action potentials [63], this may underlie possible discrepancies between the CD model and the models derived from action potential data.

### Correspondence of BOLD Signal and Neural Signals

The present study as well as the CD model is based on certain assumption on the relationship between hemodynamic and neural measures. How do we know that the sublinear summation of the BOLD response is not due to hemodynamic redistribution of blood (i.e. stealing)? Our group has probed this issue earlier by varying visual parameters in a center–surround experiment and showed that suppression had at least partially neural origin [64]. It has been known for some time that positive BOLD signal is correlated with both multiunit activity (MUA) as well as local field potentials (LFP), with somewhat better correlation between BOLD and LFP than between BOLD and MUA [62]. Recently, the negative BOLD response (NBR) has been firmly associated with reduction of neural activity from baseline [43], [65] and reduction in cerebral metabolic rate of oxygen consumption [45], [66]. While broad–band LFP in some cases may have no association with NBR [67], there is a clear association between neural hyperpolarization, i.e. inhibition of neural activity, and NBR [44]. Moreover, Devor et al. [44] showed that the NBR was due to arteriolar vasoconstriction, instead of blood stealing. Finally, Fukuda et al. [68] examined the vascular point spread function and compared cerebral blood volume changes to oxygen consumption and optical imaging of intrinsic signals. They conclude that hemodynamic volume changes are associated with a point spread which must have a smaller width than the functional columns. Thus we can be relatively confident that the increases and decreases of BOLD response reflect increases and decreases of neural activation, when we compare changes within a voxel in a balanced design. However, with the current data alone we cannot fully exclude the possibility of hemodynamic effects contributing to our results.

### Local Decorrelation

Our interaction data matched well the theoretically computed fully decorrelating modulation index *d_T_* regardless of the voxel selection, as long as the voxels represented either the center or the surround stimulus. We interpret this as an indication that the same decorrelating mechanism is applied in all visual cortical areas, and in eccentricities which represent either center or the surrounds. The main evidence supporting this claim is the same goodness–of–fit to the cortical decorrelation model in all visual areas and eccentricities. Critically, the decorrelation process, which is presumably local [33], [34], must have access to some distant neural signals in order to modulate activity for distant visual objects. We assume that the large spread of BOLD signal modulation, discussed above, reflects the long–distance access.

### Relation between Positive and Negative BOLD Response and Suppression/facilitation

Our results showed that the modulation sign (suppressive interaction or facilitative interaction) clearly depend on the relative BOLD signal sign between the two stimuli. Facilitation emerged when one of the component signals was positive and the other negative. In contrast, suppression emerged when both component signals had the same sign. Together, the decorrelation model and voxel sign dependency between activation and modulation recasts the phenomenon of suppressive interaction and facilitative interaction along a continuum, where simple network mechanisms, including the correlation between the activation patterns, determine the interaction strength and sign.

### Methodological Considerations

Our model fails in two cases: i) When the voxels comprise low average C+S values, i.e. where neither center nor surround stimulus is primarily represented. The failure of the model emerges most likely from noise. When C+S is close to noise level, the denominator of Equation 2 is close to zero and the *d_M_* value becomes unstable. ii) We also suspect that the model is inefficient when the VOI size is large and samples neurons with inhomogeneous functionality, i.e. significant amount of neurons within the VOI are sensitive to different types of input. For example when the VOI includes both facilitative and suppressive interactions, individual voxels will not be accurately predicted by our model. So far, the CD model has been evaluated only within a local population of voxels, where it has been able to account for the mean measured modulation index (*d_M_*). In a larger VOI, it is possible to include several clusters of voxels with different modulation strengths. This can be experimentally addressed by determining the kind of distribution that the the mean *d_M_* value emerges from. More specifically, do the *d_M_* values cluster around the *d_T_*, which would mean that the CD model is able to predict the pattern of activation and not only the mean modulation? It is possible that within a large VOI area, assigning one *d_T_* to the population of voxels (as is done in the CD model) makes *d_M_* values less probable to cluster around the d_T_. In the future one could try to predict a more exact sum of the component patterns (i.e. the pattern of m(C,S)) if we divide the voxels within a large VOI to multiple clusters of voxels according to the correlation values and apply Equation 3 systematically to these clusters.

In addition, the *d*–coefficient is sensitive to noise covariance. Noise that is auto-correlated in time within each voxel, adds twice to the denominator and once to the numerator in Equation 2, and consequently, biases the *d*–coefficient towards 0.5 when there is a strong temporal noise correlation in the data, In our current experimental design such temporal noise correlations were carefully accounted for by examining sham data (data points without stimulation) and constructing a design where the impact of noise correlation is minimal. We used Latin square balancing of the blocks for the first half of the runs length and mirrored the same Latin square for the second half. Paying careful attention to these issues is important for replicating our results.

Our approach is not dependent on whether a voxel summates neural activation spatially as a simple low–pass filter, or whether the filter is more complex, because of local vasculature [69], [70]. As our model is not based on any assumption about this filtering, our model should hold as long as each voxel represents the same neural population in different conditions (C, S, m(C,S)),. In theory, a voxel may sample veins, which pool BOLD signal from relatively distant neural populations (such as C and S_N_), compromising our estimates of correlation between activation patterns. This suggests that avoiding draining veins would increase the match between the model and the data.

Our data, as well as earlier results [43], [46], [48], show that negative and positive BOLD effects spread over very large distances along the visual cortex, suggesting that the neural and vascular responses have good correspondence (see the NBR discussion above). Even if the earlier estimates of BOLD point spread at 3T gradient–echo sequence (3.9 mm, [71], see however Grinvald, et al. [72] for similar neural point spread) would come from vascular spread alone, it would be less than the BOLD spread in our study.

We used generic magnification factors in Schwartz formula [47] with parameters a = 1, k = 17 in order to calculate average distance across the cortex in millimeters. As we have a large number of subjects (N = 15), it is unlikely that the generic magnification factor would be significantly different from the mean factor of our subjects. However, varying suppressive interaction at different eccentricities in the multifocal localizer may cause inaccurate voxel selection in some subjects. Averaging across subjects, however, should diminish such errors. Particularly, the spatial order of eccentricities is likely to be preserved, but there might be shifts in the mean location because of unequal suppressive interaction in the middle compared with the innermost and outermost rings, 1°–2.3° and 8.7°–12° respectively. Because the 1°–2.3° and 8.7°–12° representations in our multifocal design may have been shifted away from the 2.3°–4.0° to 6.1°–8.7° and not follow the exact retinotopic position, we may be underestimating the cortical distance between the representations of the rings. Thus, the values reported in Table 2 should be considered as the lower limits of the BOLD spread.

Attention affects BOLD responses and may create important confounding factors for interpretation of the results [73]. In our study attention was carefully controlled with a demanding letter counting task, thus attentional effects should be a significant confound in our fMRI results.

### Conclusions

The CD model aims to predict contextual modulation strength by assuming a robust sensory interaction phenomenon which reflects efficient coding [25]. Efficient coding in the CD model is achieved by decorrelation of overlapping activation patterns. This idea is not new. Response equalization by decorrelation was already reviewed by Barlow and Foldiak [26] more than 20 years ago. Conceptually, our finding is related to multivariate fMRI analysis, where information has been retrieved from the voxel activation patterns ([74]–[76]). The CD model suggests that information is not only represented macroscopically, but also modulated to account for the redundancy in large-scale activation patterns.

Our results show that strength of interaction between multiple stimuli can be predicted from the correlation of the activation patterns for the component stimuli, and thus this work is in line with earlier suggestions of decorrelation in the visual cortex [1], [21]. In addition, we found that a similar mechanism operates everywhere in the visual cortex. The interaction is supported by the brain’s connectivity, where synaptic activation and subsequent modulation can appear far from the maximum neural response. Future studies should combine physiological models and current data to find out how decorrelation is implemented at the neural level.

## Supporting Information

Figure S1 Fitting a 3^rd^ order polynomial (a–d) and linear (e–h) functions to correlation between C and S vectors versus average *d_M_*, (See Fig. 6). First data for 14 subjects was included for fitting, and one subject was left out. Then the procedure was repeated for all the subjects (dashed gray lines (a–d) and light blue lines (e–h)). The left and right columns show results for the near and far surround conditions, respectively. The voxels were selected at P_FWE_ <0.01. The black solid lines indicate the prediction (*d_T_*) from the CD model and the green (a–d) and red (e–h) solid lines indicate the group median of the 15 individual fitted functions (dashed gray lines (a–d) and light blue lines (e–h)).
(TIF)

Figure S2 The individual error between model and data at different eccentricities subtracted from error for all visual areas (VOI_AVA_). a–e) for E_SN_ (see Fig. 7 legend for details). In each VOI, the data is restricted to a resampled subset of voxels to make the errors comparable. f–j) Same for E_SF._ The error bars show the standard error of mean across all active voxels.
(TIF)

Figure S3 Same as Fig. S2 but for different functional areas. a–f) E_SN_ g–l) E_SF._
(TIF)

Table S1 Significance of BOLD signals spread and the suppressive and facilitative interactions for the Subject 3 (data points in Figure 2).“+” sign means significantly positive (sign test, p<0.05) and “–” sign means significantly negative (p<0.05) value. The test was conducted across voxels in each VOI.
(DOCX)

Table S2 As in Table S1, but for the group analysis (data points in Figure 3). The test was conducted across subjects.
(DOCX)
