# Supplementary figures and images for: Visual Interactions Conform to Pattern Decorrelation in Multiple Cortical Areas
Source: PLoS One. 2013 Jul 10;8(7):e68046. doi: 10.1371/journal.pone.0068046 (PMC3707897; doi:10.1371/journal.pone.0068046)

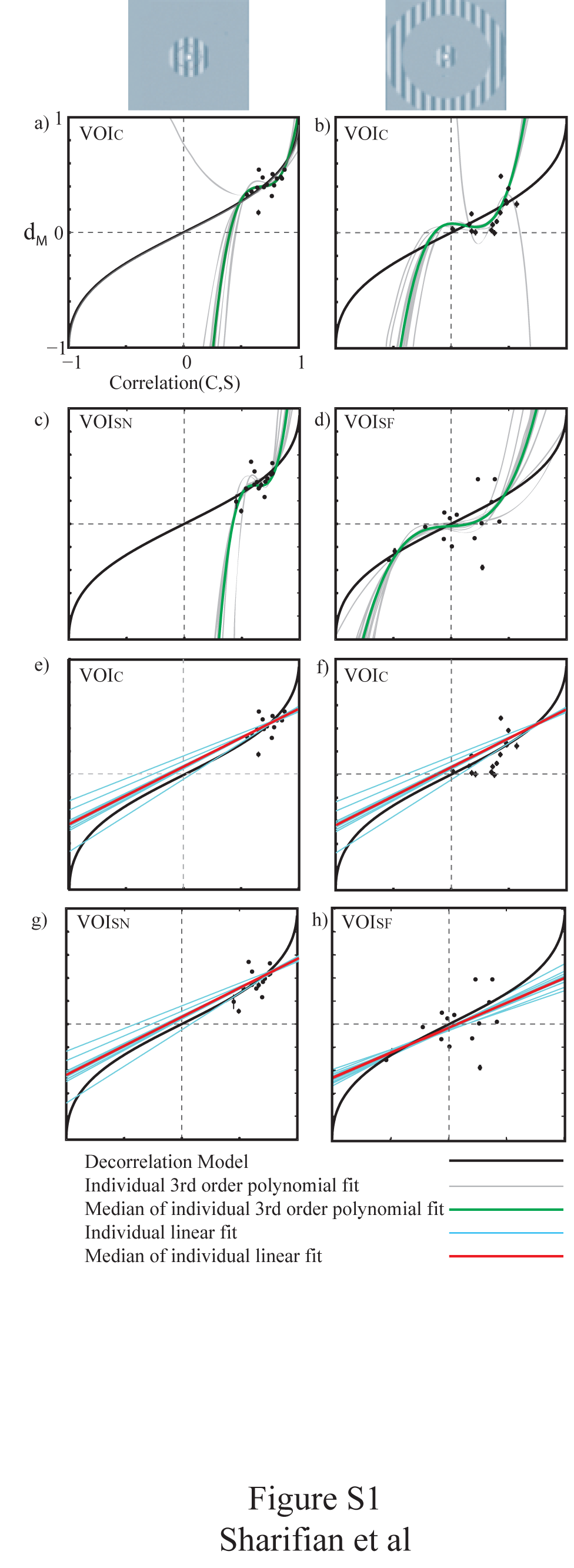

Supplement: Figure S1 — Fitting a 3rd order polynomial (a–d) and linear (e–h) functions to correlation between C and S vectors versus average dM, (See Fig. 6). First data for 14 subjects was included for fitting, and one subject was left out. Then the procedure was repeated for all the subjects (dashed gray lines (a–d) and light blue lines (e–h)). The left and right columns show results for the near and far surround conditions, respectively. The voxels were selected at PFWE <0.01. The black solid lines indicate the prediction (dT) from the CD model and the green (a–d) and red (e–h) solid lines indicate the group median of the 15 individual fitted functions (dashed gray lines (a–d) and light blue lines (e–h)). (TIF) [file pone.0068046.s001.tif]

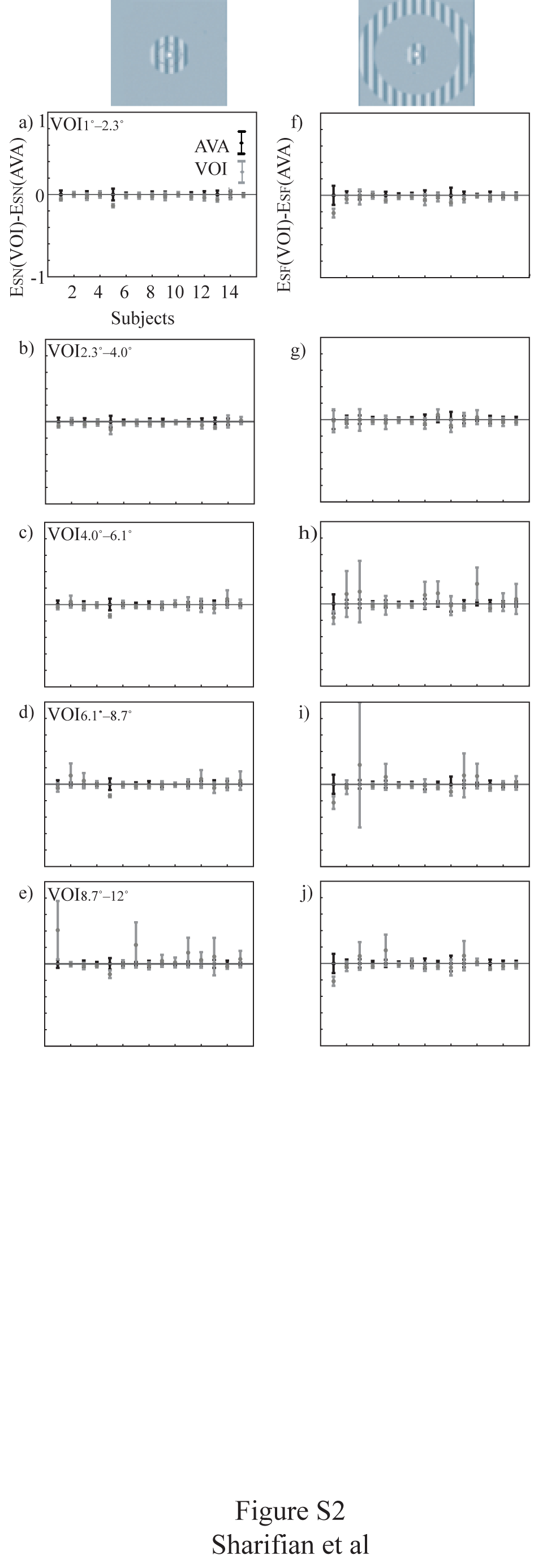

Supplement: Figure S2 — The individual error between model and data at different eccentricities subtracted from error for all visual areas (VOIAVA). a–e) for ESN (see Fig. 7 legend for details). In each VOI, the data is restricted to a resampled subset of voxels to make the errors comparable. f–j) Same for ESF. The error bars show the standard error of mean across all active voxels. (TIF) [file pone.0068046.s002.tif]

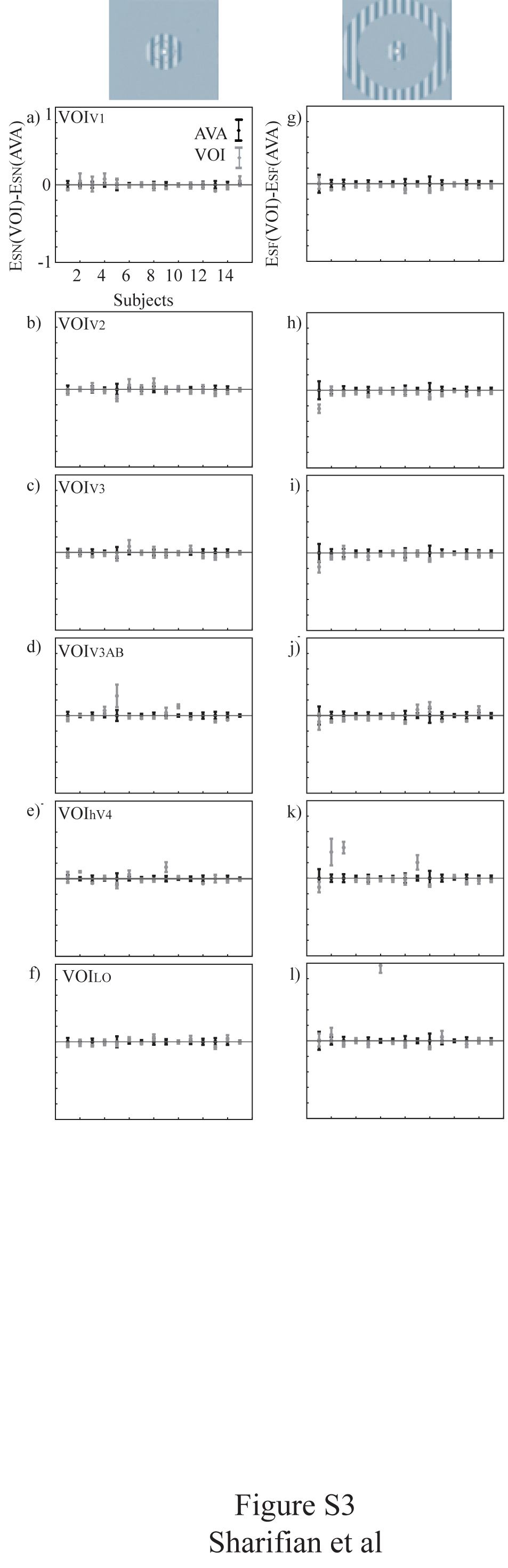

Supplement: Figure S3 — Same as Fig. S2 but for different functional areas. a–f) ESN g–l) ESF. (TIF) [file pone.0068046.s003.tif]
