# Supplementary material for: Visual Interactions Conform to Pattern Decorrelation in Multiple Cortical Areas
Source: PLoS One. 2013 Jul 10;8(7):e68046. doi: 10.1371/journal.pone.0068046 (PMC3707897; doi:10.1371/journal.pone.0068046)
Supplement: Table S1 — Significance of BOLD signals spread and the suppressive and facilitative interactions for the Subject 3 (data points in Figure 2).“+” sign means significantly positive (sign test, p<0.05) and “–” sign means significantly negative (p<0.05) value. The test was conducted across voxels in each VOI. (DOCX) [file pone.0068046.s004.docx]

Significance of BOLD signals spread and interactions

|  | **V1** | | | | | | | | | | |
| --- | --- | --- | --- | --- | --- | --- | --- | --- | --- | --- | --- |
|  | **Near surround** | | | | |  | **Far surround** | | | | |
|  | 1˚–2.3˚ | 2.3˚–4.0˚ | 4.0˚–6.1˚ | 6.1˚–8.7˚ | 8.7˚–12˚ |  | 1˚–2.3˚ | 2.3˚–4.0˚ | 4.0˚–6.1˚ | 6.1˚–8.7˚ | 8.7˚–12˚ |
| C | + | + | - | - | - |  | + | + | - | - | - |
| S | + | + | + |  | - |  | - |  |  | + | + |
| m(C,S) | + | + |  | - | - |  | + | + |  | + | + |
| C+S | + | + |  | - | - |  | + | + |  |  | + |
| m(C,S)-(C+S) | - | - |  |  |  |  | + | + | + | + | + |
|  | **V2** | | | | | | | | | | |
|  | **Near surround** | | | | |  | **Far surround** | | | | |
|  | 1˚–2.3˚ | 2.3˚–4.0˚ | 4.0˚–6.1˚ | 6.1˚–8.7˚ | 8.7˚–12˚ |  | 1˚–2.3˚ | 2.3˚–4.0˚ | 4.0˚–6.1˚ | 6.1˚–8.7˚ | 8.7˚–12˚ |
| C | + | + | - | - | - |  | + | + | - | - | - |
| S | + | + | + |  | - |  | - | + |  | + | + |
| m(C,S) | + | + | + |  | - |  | + | + | + | + | + |
| C+S | + | + | + |  | - |  | + |  |  | + | + |
| m(C,S)-(C+S) | - | - |  |  | + |  | + | + | + |  | + |
|  | **V3** | | | | | | | | | | |
|  | **Near surround** | | | | |  | **Far surround** | | | | |
|  | 1˚–2.3˚ | 2.3˚–4.0˚ | 4.0˚–6.1˚ | 6.1˚–8.7˚ | 8.7˚–12˚ |  | 1˚–2.3˚ | 2.3˚–4.0˚ | 4.0˚–6.1˚ | 6.1˚–8.7˚ | 8.7˚–12˚ |
| C | + | + |  | + |  |  | + | + |  | + |  |
| S |  | + | + | + |  |  | - |  |  | + | + |
| m(C,S) | + | + | + | + |  |  | + | + | + | + | + |
| C+S | + | + | + | + |  |  | + | + |  | + | + |
| m(C,S)-(C+S) |  | - | - |  |  |  | + |  |  |  |  |

Table S1

Sharifian et al
