# Supplementary material for: Visual Interactions Conform to Pattern Decorrelation in Multiple Cortical Areas
Source: PLoS One. 2013 Jul 10;8(7):e68046. doi: 10.1371/journal.pone.0068046 (PMC3707897; doi:10.1371/journal.pone.0068046)
Supplement: Table S2 — As in Table S1, but for the group analysis (data points in Figure 3). The test was conducted across subjects. (DOCX) [file pone.0068046.s005.docx]

Significance of BOLD signals spread and interactions

|  | **V1** | | | | | | | | | | |
| --- | --- | --- | --- | --- | --- | --- | --- | --- | --- | --- | --- |
|  | **Near surround** | | | | |  | **Far surround** | | | | |
|  | 1˚–2.3˚ | 2.3˚–4.0˚ | 4.0˚–6.1˚ | 6.1˚–8.7˚ | 8.7˚–12˚ |  | 1˚–2.3˚ | 2.3˚–4.0˚ | 4.0˚–6.1˚ | 6.1˚–8.7˚ | 8.7˚–12˚ |
| C | **+** | **+** |  |  | **-** |  | **+** | **+** |  |  | **-** |
| S | **+** | **+** | **+** | **+** |  |  | **+** | **+** | **+** | **+** | **+** |
| m(C,S) | **+** | **+** | **+** | **+** |  |  | **+** | **+** | **+** | **+** | **+** |
| C+S | **+** | **+** | **+** |  | **-** |  | **+** | **+** | **+** | **+** | **+** |
| m(C,S)-(C+S) | **-** | **-** |  |  |  |  |  |  |  |  |  |
|  | **V2** | | | | | | | | | | |
|  | **Near surround** | | | | |  | **Far surround** | | | | |
|  | 1˚–2.3˚ | 2.3˚–4.0˚ | 4.0˚–6.1˚ | 6.1˚–8.7˚ | 8.7˚–12˚ |  | 1˚–2.3˚ | 2.3˚–4.0˚ | 4.0˚–6.1˚ | 6.1˚–8.7˚ | 8.7˚–12˚ |
| C | **+** | **+** | **+** |  | **-** |  | **+** | **+** | **+** |  | **-** |
| S | **+** | **+** | **+** | **+** |  |  |  | **+** | **+** | **+** | **+** |
| m(C,S) | **+** | **+** | **+** | **+** | **-** |  | **+** | **+** | **+** | **+** | **+** |
| C+S | **+** | **+** | **+** | **+** | **-** |  | **+** | **+** | **+** | **+** | **+** |
| m(C,S)-(C+S) | **-** | **-** | **-** |  |  |  |  |  |  |  | **+** |
|  | **V3** | | | | | | | | | | |
|  | **Near surround** | | | | |  | **Far surround** | | | | |
|  | 1˚–2.3˚ | 2.3˚–4.0˚ | 4.0˚–6.1˚ | 6.1˚–8.7˚ | 8.7˚–12˚ |  | 1˚–2.3˚ | 2.3˚–4.0˚ | 4.0˚–6.1˚ | 6.1˚–8.7˚ | 8.7˚–12˚ |
| C | **+** | **+** |  |  |  |  | **+** | **+** |  |  |  |
| S | **+** | **+** |  |  |  |  |  | **+** |  |  | **+** |
| m(C,S) | **+** | **+** |  |  |  |  | **+** | **+** |  |  | **+** |
| C+S | **+** | **+** |  |  |  |  | **+** | **+** |  |  | **+** |
| m(C,S)-(C+S) | **-** | **-** | **-** | **-** | **-** |  |  | **-** |  | **-** |  |

Table S2

Sharifian et al
